# Impact of Individual Colonic Segment Histological Activity on Disease Relapse in Patients with Ulcerative Colitis

**DOI:** 10.3390/jcm14144962

**Published:** 2025-07-13

**Authors:** Steven Li Fraine, Victoria Marcus, Chelsea Maedler Kron, Peter L. Lakatos, Waqqas Afif, Alain Bitton, Gary Wild, Talat Bessissow

**Affiliations:** 1 Inflammatory Bowel Disease Centre, Division of Gastroenterology and Hepatology, McGill University Health Centre (MUHC), Montreal, QC H4A-3J1, Canadakislakpet99@gmail.com (P.L.L.); waqqas.afif@mcgill.ca (W.A.); alain.bitton.med@ssss.gouv.qc.ca (A.B.);; 2 Department of Pathology, McGill University Health Centre (MUHC), Montreal, QC H4A-3J1, Canada; 3 First Department of Medicine, Semmelweis University, 1085 Budapest, Hungary

**Keywords:** ulcerative colitis, histology, Geboes, relapse

## Abstract

**Background/Objectives**: The aim of this study was to assess the role of histological activity in individual segments of the colon in predicting disease relapse in patients with ulcerative colitis. **Methods**: This was a prospective observational study on patients with ulcerative colitis in clinical remission. Biopsies were taken of multiple segments of the colon, and histological activity was assessed using the Geboes (GB) score. Patients were monitored for disease relapse for 12 months. The primary objective was to determine the predictive value of histological activity of the individual segments of the colon on disease relapse. The secondary objective was to assess whether having multiple segments in histological remission is associated with disease relapse. **Results**: Of 253 patients, 19% had disease relapse. Histological activity (GB ≥ 3.1) was not predictive of disease relapse for the rectum (adjusted odds ratio [aOR] 0.95, confidence interval [CI] 0.46–1.98, *p* = 0.894), sigmoid (aOR 0.67, CI 0.24–1.90, *p* = 0.451), descending colon (aOR 1.52, CI 0.43–5.39, *p* = 0.519), transverse colon (aOR 0.47, CI 0.10–2.18, *p* = 0.332), and right colon (aOR 1.75 CI 0.73–4.18, *p* = 0.209). Histological remission (GB ≤ 2.0) was also not predictive of remaining in remission for any individual colonic segment nor was there any benefit of having multiple segments with histological remission compared to having ≤1 segment in histological remission (aOR 0.56, CI 0.28–1.10, *p* = 0.093). **Conclusions**: Histological activity in any individual colonic segment or the number of colonic segments with histological remission was not predictive of disease relapse.

## 1. Introduction

Ulcerative colitis (UC) is a form of idiopathic chronic inflammatory bowel disease (IBD) in which there is continuous mucosal inflammation of the colon from the rectum and extending proximally. The disease is characterized by an unpredictable relapsing and remitting course and can lead to serious complications including bleeding, megacolon, perforation, and cancer [1,2,3]. Current international guidelines support endoscopic healing as the preferred long-term treatment goal for patients with UC as it is associated with improved outcomes including surgery-free survival, corticosteroid-free clinical remission, lower risk of cancer, and improved quality of life [1,4,5,6,7,8,9]. Targeting endoscopic healing has become significantly more feasible in recent years, predominantly due to the introduction of novel medical therapies such as biologics and oral small molecules [9].

Many experts foresee that histological healing will be the future endpoint for patients with UC. This stems from previous studies showing an association between histological activity and relapse rates [10], whereas histological healing has been associated with a reduction in hospitalization and need for surgery [11,12]. In fact, there are multiple studies to suggest that histological activity may be present in up to 40% of patients with UC despite having endoscopic healing [12,13,14,15]. Some studies, however, suggest that histological remission does not influence relapse in patients with UC who are in endoscopic remission [16]. The Geboes (GB) score is an objective measurement of histological activity that shows good interobserver agreement [17].

In a previous prospective observational study, our group found that the overall histological activity using the Geboes score did not significantly impact the time to clinical relapse [1]. These findings were limited to the histological activity of a single colonic segment. In clinical practice, a limited flexible sigmoidoscopy is sometimes performed to assess for endoscopic activity and response to treatment. This method is faster, requires less preparation, and can be more easily used without any form of sedation for the patient when compared to a full colonoscopy. It is unclear, however, whether evaluating the histological activity of the more proximal colon has any clinical benefit. Knowing the role of histological activity in different segments of the colon may help determine whether a full colonoscopy, as opposed to a limited flexible sigmoidoscopy, is warranted to help stratify patients for the risk of relapse.

The primary objective of this novel extension study was to determine the predictive value of histological activity of the individual segments of the colon on disease relapse in patients with UC during a 1-year follow-up. The secondary objective was to assess the relevance of the number of colonic segments with histological remission on disease relapse.

## 2. Materials and Methods

### 2.1. Study Population and Design

We performed a prospective observational study at the McGill University Health Centre IBD clinic between July 2012 and July 2020 that extends from our previous study in 2022 [1]. All patients 18 years and older with a confirmed diagnosis of UC in clinical remission for at least 3 months undergoing colonoscopy for disease assessment were included in this study. Diagnosis of UC was based on accepted clinical, endoscopic, and histological criteria. Clinical remission was defined as a partial Mayo score of ≤2 points with a rectal bleeding score of zero. Exclusion criteria included those with previous surgical resection, indeterminate colitis, disease remission over 10 years, use of oral or rectal steroids within 90 days of study entry, and altered dosage of oral 5-ASA, thiopurine, or anti-tumour necrosis factor α within 3 months before study entry.

### 2.2. Histological Assessment

During colonoscopy, biopsies were taken throughout the colon per clinician judgement. Individual segments were biopsied at the discretion of the physician. Samples were categorized into colonic segments (right colon [included ascending colon and cecum], transverse colon, descending colon, sigmoid colon, and rectum) based on landmarks identified during endoscopy. In certain instances, samples were labelled based on distance from insertion site (in cm). When the segment was not labelled, for the purpose of the analysis, the samples were re-classified into colonic segments as follows: right colon (70 to 100 cm), transverse colon (50 to 69 cm), descending colon (40 to 49 cm), sigmoid (20 to 39 cm), and rectum (<20 cm). All samples were immediately fixed in 10% neutral formalin, processed, and then sections were stained with hematoxylin and eosin. All samples were independently examined by one of two gastrointestinal pathologists, blinded to any relevant clinical information. A histological grade was given using the Geboes score which evaluates structural changes, inflammatory cell infiltration, crypt destruction, and erosions or ulceration [18]. If multiple biopsy samples of a given colonic segment were available, the higher histological grade was used. Patients were assessed every 3 months over a 12-month period or earlier if they developed clinical symptoms suggestive of disease relapse. A sigmoidoscopy was performed to confirm endoscopic activity of active disease (endoscopic Mayo score of 2 or 3). All patients underwent endoscopic assessment at the end of the 12-month follow-up to document the disease activity.

### 2.3. Study Variables

Patient demographics and disease characteristics including age, sex, smoking status, extent of disease, number of relapses in the last 2 years, and medical therapy at the time of recruitment and at follow up were collected at baseline from chart review and direct patient interview. Clinical relapse was defined as a full Mayo score ≥3. Histological activity was assessed using the Geboes score [18]. We defined active histological disease as a Geboes score of ≥3.1 and histological remission as a score of ≤2.0.

### 2.4. Statistical Analysis

Research Ethics Board approval was obtained from the McGill University Health Centre. All data was collected via chart review. Patient information was kept confidential. Descriptive analysis (mean, median, and interquartile range (IQR) for continuous data or percentage for dichotomous data) were performed. Unadjusted and adjusted odds ratios (ORs) and 95% confidence intervals (CIs) were used to predict relapse with respect to Geboes scores in each colonic segment as well for the number of colonic segments using logistic regression analysis. Time-event analysis of 1-year relapse using Kaplan–Meier curves and Cox regression analysis were performed. All odds ratios and hazard ratios were adjusted for baseline age, sex, smoking history, extent of disease, and treatment regimen. A *p*-value < 0.05 was considered statistically significant for all results. Statistical analysis was performed using SPSS statistical software package version 29.0 (IBM, New York, NY, USA).

## 3. Results

### 3.1. Baseline Characteristics

A total of 253 patients with UC in clinical remission at the time of colonoscopy were included in this study. Baseline characteristics are included in Table 1. The median age at the time of colonoscopy was 47 years old with close to half of the patients being female (46.2%). A minority of patients had a history of smoking (14.3%). Most patients had either left-sided or extensive disease. The median duration of disease was 13 years. Approximately half (48.4%) of patients did not have a disease relapse in the last two years. The majority (68.4%) of patients were on a 5-ASA, and 19.0% of patients were on a biologic. There were 26 (10.3%) patients who had histological activity (GB ≥ 3.1) more proximal than mucosal activity.

### 3.2. Geboes Score as Predictor of Clinical Relapse

After a 1-year follow-up, 48 (19%) patients developed clinical relapse of their disease. All patients with clinical signs of relapse were confirmed to have an endoscopic Mayo score of ≥2 and, thus, the confirmation of endoscopic activity. Of all enrolled patients, 93.7% had biopsies of the rectum available for calculation of the histological Geboes score compared to 64.8% with sigmoid colon, 40.3% with descending colon, 51.4% with transverse colon, and 74.3% with right colon biopsies. When comparing a baseline Geboes score of ≥3.1 (active histology) with a score of <3.1, there was no increase in the risk of clinical relapse for the rectum with adjusted odds ratio (OR) = 0.95 (CI 0.46–1.98, *p* = 0.894), sigmoid colon with adjusted OR = 0.67 (CI 0.24–1.90, *p* = 0.451), descending colon with adjusted OR = 1.52 (CI 0.43–5.39, *p* = 0.519), transverse colon with adjusted OR = 0.47 (CI 0.10–2.18, *p* = 0.332), and right colon with adjusted OR = 1.75 (CI 0.73–4.18, *p* = 0.209). Furthermore, a baseline Geboes score of ≤ 2.0 was not associated with a decreased risk of relapse for any of the colonic segments (rectum adjusted OR = 0.61 (CI 0.31–1.20, *p* = 0.250), sigmoid colon adjusted OR = 0.95 (CI 0.47–1.89, *p* = 0.875), descending colon adjusted OR = 0.51 (CI 0.22–1.76, *p* = 0.114), transverse colon adjusted OR = 0.69 (CI 0.33–1.47, *p* = 0.340), and right colon adjusted OR = 0.68 (CI 0.34–1.35, *p* = 0.266; Table 2)). Given that some segments had low sampling rates, a sensitivity analysis was performed, including only patients in whom at least three segments were biopsied. A Geboes score of ≤2.0 in the descending colon was associated with a lowered risk of relapse with adjusted OR = 0.34 (CI 0.21–0.98, *p* = 0.045). No other segments had an increased or decreased risk of relapse in relation to Geboes scores (Supplemental Table S1).

**Figure 1:**
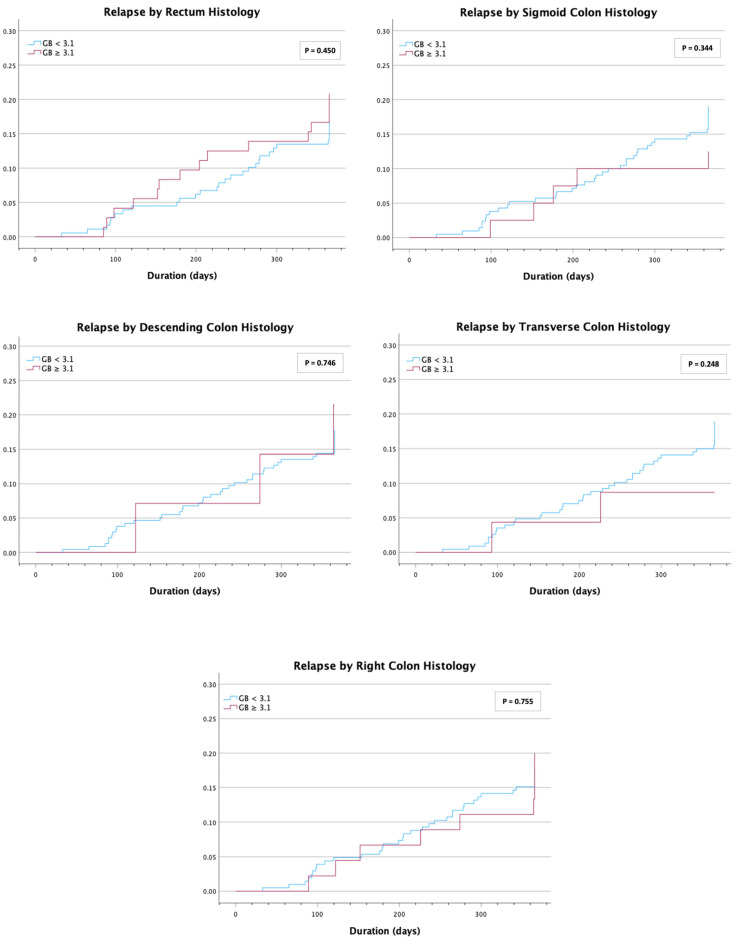
Proportion of patients with disease relapse over time for individual colonic segments with a baseline Geboes score (GB) < 3.1 vs. ≥3.1.

**Figure 2:**
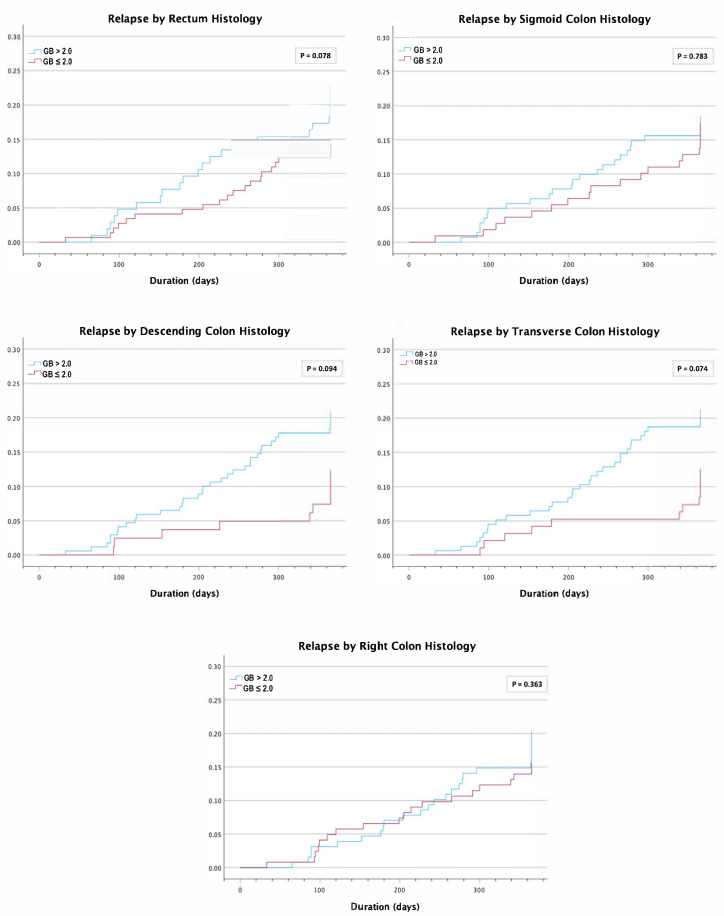
Proportion of patients with disease relapse over time for individual colonic segments with a baseline Geboes score (GB) > 2.0 vs. GB ≤ 2.0.

A time-event analysis using a Cox regression model and Kaplan–Meier curves (Figure 1) showed no difference in risk of relapse with a GB ≥3.1 for the rectum with hazard ratio (HR) = 1.27 (CI 0.68–2.36, *p* = 0.453), sigmoid colon with HR = 0.64 (CI 0.25–1.63, *p* = 0.349), descending colon with HR = 1.21 (CI 0.38–3.91, *p* = 0.747), transverse colon with HR = 0.45 (CI 0.11–1.84, *p* = 0.263), and right colon with HR = 1.12 (CI 0.54–2.33, *p* = 0.756). Similarly, there was no difference in risk of relapse with a GB ≤ 2.0 (Figure 2) for the rectum with hazard ratio (HR) = 0.60 (CI 0.33–1.07, *p* = 0.082), sigmoid colon with HR = 0.92 (CI 0.51–1.66, *p* = 0.783), descending colon with HR = 0.55 (CI 0.27–1.12, *p* = 0.100), transverse colon with HR = 0.55 (CI 0.29–1.07, *p* = 0.079), and right colon with HR = 0.76 (CI 0.42–1.38, *p* = 0.366).

**Figure 3:**
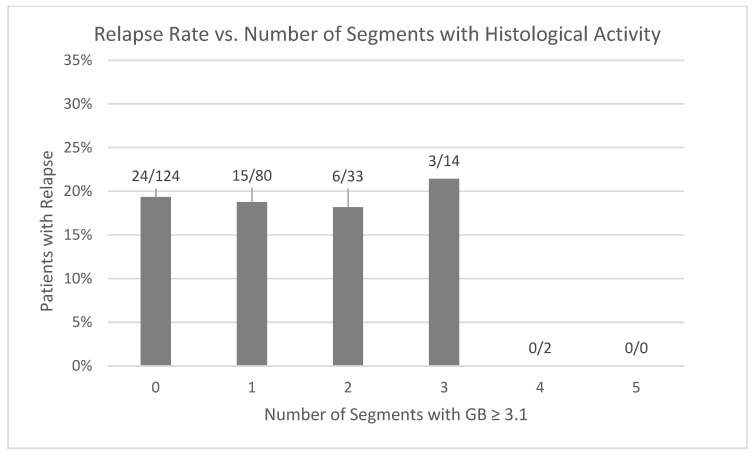
Relapse rates depending on the total number of segments with histological activity (GB ≥ 3.1). No patients with 4 or 5 segments with histological activity have disease relapse.

**Figure 4:**
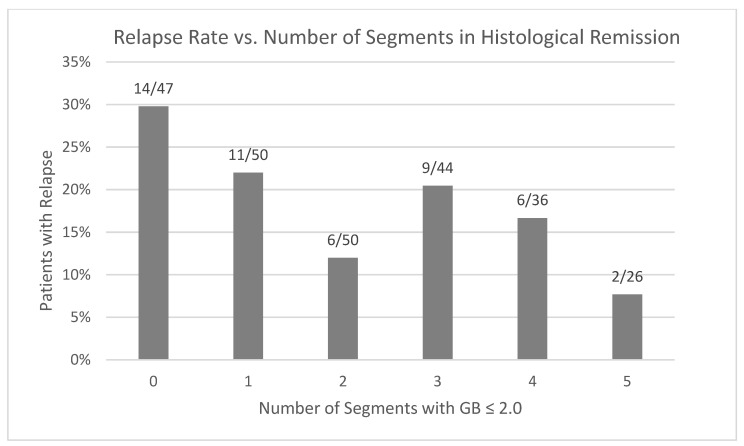
Relapse rates depending on the total number of segments with histological remission (GB ≤ 2.0).

**Figure 5:**
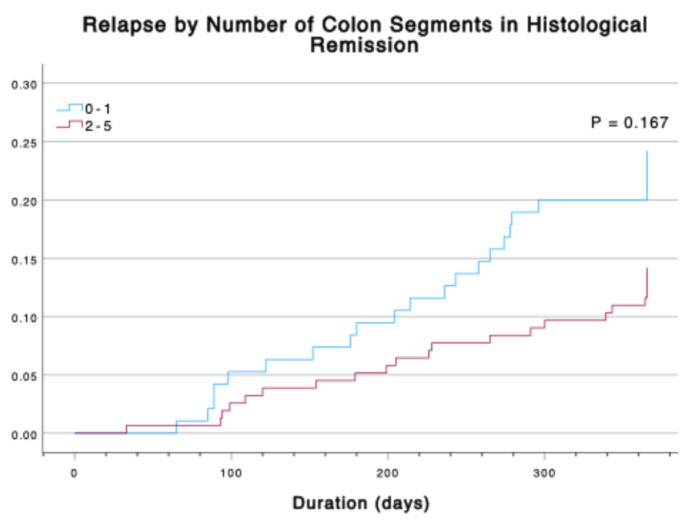
The 1-year relapse rates for 0–1 vs. 2–5 colonic segments with baseline histological remission (GB ≤ 2.0).

The risk of relapse was measured in comparison to the number of colonic segments in histological remission. The number of segments with histological activity (Figure 3) or histological remission (Figure 4) was not associated with a risk of disease relapse after one year. No patients with four or five segments with histological activity had disease relapse after one year; however, only two patients total had four segments with a GB ≥ 3.1, and no patients had five segments with a GB ≥ 3.1. Furthermore, having multiple segments in histological remission was not associated with a lower risk of clinical relapse compared to having one or zero segments with an adjusted OR = 0.56 (CI 0.28–1.10, *p* = 0.093). A time-event analysis with a Cox regression model and Kaplan–Meier curves (Figure 5) also demonstrates no decrease in risk of relapse when having at least two segments with histological remission as compared to having zero or one segments with adjusted HR = 0.645 (CI 0.35–1.20, *p* = 0.167).

## 4. Discussion

The relevance of histological activity in ulcerative colitis stems from multiple studies revealing that up to 40% of patients with UC in clinical and endoscopic remission still had histological activity [12,13,14,15,19]. In recent years, there has been a push in the literature that histological remission should be a more stringent treatment target as compared to mucosal healing, as it may represent a more complete marker of disease activity. However, there is not yet a clear consensus on its relevance [9,16]. Furthermore, understanding the role of histological activity in different segments of the colon can help guide whether a full colonoscopy is necessary compared to a flexible sigmoidoscopy in risk-stratifying patients for disease relapse.

Our group previously found that active histology with a Geboes score of ≥3.1 was not associated with predicting disease in patients with UC [1]. In this study, we looked to see if the histological activity of the individual segments of the colon using the Geboes score was associated with clinical relapse within a 12-month follow-up. Indeed, active histology as defined by a Geboes score ≥ 3.1 in any segment of the colon was not associated with disease relapse. Furthermore, the European Crohn’s and Colitis Organization (ECCO) defines histological remission as a Geboes score of less than grade 2 [20,21]. In this present study, a Geboes score of ≤2.0 in an individual colonic segment was similarly not associated with a decrease in clinical relapse. Our study contrasts with other prospective studies, including two in which a baseline Geboes score of ≥3.1 in patients with UC in endoscopic remission had an increased risk of developing clinical relapse within a 12-month period [12,13]. One prospective study by Cushing et al. showed that the lack of histological normalization defined by a Geboes score > 0 was associated with an increased likelihood of relapse [22]. However, it is unclear what the clinical significance histological normalization (GB = 0) is compared to histological remission (GB ≤ 2.0). Their study showed no association between Geboes scores of ≥2.1 and ≥3.1 [22]. Studies such as that by Bryant et al. showed that histological remission was predictive of clinical outcomes; however, the Truelove and Richard’s index was used as a histological grading system, which is not as well validated as the Geboes score [14]. In contrast, Zenlea et al. showed a strong association between clinical outcomes and histological grades using the Geboes score; however, relapse was not confirmed by endoscopic evaluation [12]. Retrospective studies have also shown an increased risk of neoplasia and relapse with active histology [8,13,23]. Another large retrospective by Narula et al. supports histological activity showing no influence on the time to clinical relapse in patients with UC [16].

Given that UC is a disease that includes the rectum and extends proximally at varying lengths, it would be expected that histological activity in this region would be most predictive of disease relapse with the role of histological activity in the proximal colon being relatively unclear. In this study, active histology in the rectum defined by a GB ≥ 3.1 was not predictive of disease relapse in UC within a 12-month follow-up with an adjusted odds ratio = 0.95 (CI 0.46–1.98, *p* = 0.894). The histological activity in more proximal segments, including the sigmoid colon, descending colon, transverse colon, and right colon, was similarly not predictive of disease relapse. Moreover, the number of segments with histological remission did not lower the risk of 1-year relapse. These findings suggest a lack of benefit in obtaining rectal biopsies or samples of more proximal segments of the colon to assess disease control. There is otherwise scarce data for the role of histological activity in individual segments of the colon. Prior studies have shown that, in patients with pancolitis, histological improvement tended to occur in the right colon before it occurred in the left and that isolated histological activity of the right colon was rare in UC [24,25]. In a retrospective study by Christensen, the complete histological normalization of all segments of the bowel was associated with a decrease in disease relapse, whereas the histological normalization of a single bowel segment was not associated with improvement in relapse-free survival [24]. Importantly, the histological activity scale used in their study has not been independently validated [24]. This data supports our finding that histological remission in a single colonic segment is not associated with a decrease in relapse risk; however, our study differs in that we do not see any benefit in obtaining complete histological normalization throughout the bowel. To the best of our knowledge, our study is the first to look at the role of the histological activity in individual bowel segments using a validated histological scoring system.

There are several strengths to this study. For one, it is a relatively large study with a total follow-up of 1 year. Patients were closely followed with baseline and 1-year follow-up colonoscopies in addition to endoscopic evaluation when relapse was suspected. Furthermore, histological samples were scored using the validated Geboes score. This study is one of the few that evaluates the histological activity of the entire colon in individual segments, which is a relevant investigation given the increasing emphasis on the importance of histological remission. This study has a few limitations. It was conducted at a single tertiary centre, which may limit the generalizability of the findings. This study design is an observational one and therefore prone to confounding and selection bias. Regarding data collection, biopsies were taken in individual segments of the colon per clinician discretion, which also adds selection bias. Rectal biopsies were taken in 93.7% of cases; however, not all patients had biopsies taken of each segment of the colon. Therefore, a more thorough assessment regarding the role of histological activity in individual colonic segments on disease relapse was not feasible during this study. A sensitivity analysis looking at only individuals with at least three segments sampled showed that a Geboes score of ≤2.0 in the descending colon was associated with a lowered risk of relapse, suggesting that the low sampling rate may skew results. Moreover, in our study, only two patients had four segments with histological activity, and no patients had five segments with histological activity. A higher sampling rate would presumably result in higher relapse rates in patients with four or five segments with histological activity. The endoscopies were conducted by five IBD gastroenterologists; however, this may lead to interobserver variability with regard to endoscopic activity as well as the accuracy of location from which the biopsy samples were taken. Finally, it remains possible that 12 months is not a long enough study period to ascertain an association between histological activity and relapse in individual segments of the colon. Therefore, future studies may require more extensive data collection.

## 5. Conclusions

In summary, this study suggests that the histological activity assessed using the Geboes score in individual segments of the colon is not predictive of disease relapse. Furthermore, there was no benefit to be found in achieving multiple segments with histological remission. These findings therefore do not suggest the use of histological activity as a new treatment target for patients with ulcerative colitis. Moreover, there is little benefit in sampling the entire colon when risk stratifying patients for future disease relapse. Further prospective studies will need to be completed to better define this role.

## Figures and Tables

**Table 1:** Baseline demographic data.

Variables	Total Cohort *N* = 253 (%)
Median age at study entry {IQR}	47 {36–58}
Female	117 (46.2)
Smokers	36 (14.3)
Extent of disease *	
Proctitis [E1]	37 (14.6)
Left-sided colitis [E2]	78 (30.8)
Extensive colitis [E3]	117 (46.2)
Median duration of disease in years [IQR]	13 {5–21}
Number of relapses—last 2 years	
0	122 (48.4)
1	87 (34.5)
≥2	43 (17.1)
Treatment	
Oral 5-ASA	173 (68.4)
Thiopurine	53 (20.9)
Infliximab	33 (13.0)
Adalimumab	9 (3.6)
Vedolizumab	6 (2.4)
Sampling rate per segment	
Rectum	237 (93.7)
Sigmoid colon	164 (64.8)
Descending colon	102 (40.3)
Transverse colon	130 (51.4)
Right colon	188 (74.3)

IQR, interquartile range. * Extent of disease per the Montreal classification.

**Table 2:** Segmental Geboes scores predicting 1-year cumulative risk of disease relapse.

Variable	Unadjusted OR (95% CI)	*p*-Value	Adjusted OR (95% CI)	*p*-Value
Rectum				
GB ≥ 3.1	1.18 (0.60–2.34)	0.634	0.95 (0.46–1.98)	0.894
GB ≤ 2.0	0.54 (0.29–1.02)	0.058	0.61 (0.31–1.20)	0.250
Sigmoid Colon				
GB ≥ 3.1	0.57 (0.21–1.53)	0.260	0.67 (0.24–1.90)	0.451
GB ≤ 2.0	0.91 (0.48–1.73)	0.779	0.95 (0.47–1.89)	0.875
Descending Colon				
GB ≥ 3.1	1.60 (0.49–5.27)	0.437	1.52 (0.43–5.39)	0.519
GB ≤ 2.0	0.50 (0.23–1.06)	0.069	0.51 (0.22–1.76)	0.114
Transverse Colon				
GB ≥ 3.1	0.38 (0.09–1.68)	0.203	0.47 (0.10–2.18)	0.332
GB ≤ 2.0	0.55 (0.27–1.09)	0.088	0.69 (0.33–1.47)	0.340
Right Colon				
GB ≥ 3.1	1.24 (0.56–2.71)	0.597	1.75 (0.73–4.18)	0.209
GB ≤ 2.0	0.65 (0.34–1.23)	0.185	0.68 (0.34–1.35)	0.266

CI, confidence interval; GB, Geboes score; and OR, odds ratio. *p*-value statistically significant at <0.05.

## Data Availability

The original contributions presented in this study are included in the Supplementary Material. Further inquiries can be directed to the corresponding author.

## Supplementary Materials

The following supporting information can be downloaded at https://www.mdpi.com/article/10.3390/jcm14144962/s1, Table S1: Segmental Geboes scores predicting 1-year cumulative risk of disease relapse in cases where ≥3 segments were biopsied.
