# Supplementary material for: Impact of Individual Colonic Segment Histological Activity on Disease Relapse in Patients with Ulcerative Colitis
Source: J Clin Med. 2025 Jul 13;14(14):4962. doi: 10.3390/jcm14144962 (PMC12295168; doi:10.3390/jcm14144962)
Supplement: Supplementary file 1 [file jcm-14-04962-s001.zip › jcm-3721111-supplementary.pdf]

**Supplementary Table S1.** Segmental Geboes scores predicting 1-year cumulative risk of disease relapse in cases where ≥ 3 segments were biopsied

| Variable | Unadjusted OR (95% CI) | P-value | Adjusted OR (95% CI) | P-value |
|---|---|---|---|---|
| Rectum | | | | |
| GB ≥ 3.1 | 1.06 (0.43-2.60) | 0.902 | 0.86 (0.32-2.32) | 0.760 |
| GB ≤ 2.0 | 0.48 (0.21-1.07) | 0.073 | 0.49 (0.20-1.22) | 0.125 |
| Sigmoid Colon | | | | |
| GB ≥ 3.1 | 0.68 (0.24-1.92) | 0.463 | 0.65 (0.21-1.99) | 0.446 |
| GB ≤ 2.0 | 1.18 (0.51-2.73) | 0.705 | 0.98 (0.39-2.49) | 0.967 |
| Descending Colon | | | | |
| GB ≥ 3.1 | 1.82 (0.54-6.17) | 0.338 | 1.54 (0.41-5.78) | 0.524 |
| GB ≤ 2.0 | 0.48 (0.21-1.11) | 0.087 | 0.34 (0.21-0.98) | 0.045 |
| Transverse Colon | | | | |
| GB ≥ 3.1 | 0.41 (0.09-1.84) | 0.241 | 0.44 (0.09-2.16) | 0.314 |
| GB ≤ 2.0 | 0.57 (0.25-1.28) | 0.171 | 0.66 (0.25-1.66) | 0.377 |
| Right Colon | | | | |
| GB ≥ 3.1 | 1.58 (0.67-3.73) | 0.297 | 2.09 (0.79-5.53) | 0.136 |
| GB ≤ 2.0 | 0.72 (0.32-1.61) | 0.418 | 0.56 (0.22-1.40) | 0.213 |

CI, confidence interval; GB, Geboes score; OR, odds ratio

P-value statistically significant at < 0.05
